# (*E*)-2-[2-(Penta­fluoro­phen­yl)ethen­yl]-8-quinolyl acetate

**DOI:** 10.1107/S1600536809043888

**Published:** 2009-10-28

**Authors:** Li-Yan Zhang, Yan-Ping Huo

**Affiliations:** a Department of Chemistry, Huangshan University, Huangshan 245041, People’s Republic of China; b Faculty of Chemical Engineering and Light Industry, Guangdong University of Technology, Guangzhou 510006, People’s Republic of China; c Key Laboratory of Organofluorine Chemistry, Shanghai Institute of Organic Chemstry, Chinese Academy of Sciences, Shanghai 200032, People’s Republic of China

## Abstract

The title compound, C_19_H_10_F_5_NO_2_, was synthesized by the 1:1 condensation of 2-methyl-8-hydroxy­quinaldine with penta­fluoro­benzaldehyde. It crystallizes with two almost identical mol­ecules in the asymmetric unit. The penta­fluoro­benzene ring is essentially coplanar with the quinoline ring, forming dihedral angles of 2.49 (17) and 8.72 (16)° in the two mol­ecules.

## Related literature

For a recent review on the synthesis of quinoline derivatives, see: Zeng *et al.* (2006 ▶).
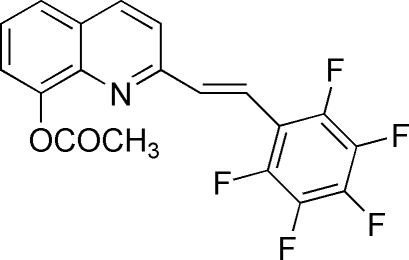


## Experimental

### 

#### Crystal data

C_19_H_10_F_5_NO_2_
*M* *_r_* = 379.28
Monoclinic, 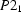

*a* = 12.3149 (13) Å
*b* = 8.6730 (9) Å
*c* = 15.0491 (16) Å
β = 93.786 (2)°
*V* = 1603.8 (3) Å^3^
*Z* = 4
Mo *K*α radiation
μ = 0.14 mm^−1^
*T* = 293 K
0.40 × 0.37 × 0.23 mm

#### Data collection

Bruker SMART CCD area-detector diffractometer
Absorption correction: multi-scan (*SADABS*; Bruker, 2001 ▶) *T*_min_ = 0.946, *T*_max_ = 0.968
9498 measured reflections
3695 independent reflections
2952 reflections with *I* > 2σ(*I*)
*R*_int_ = 0.041

#### Refinement

*R*[*F* ^2^ > 2σ(*F* ^2^)] = 0.043
*wR*(*F* ^2^) = 0.108
*S* = 0.98
3695 reflections
490 parameters
1 restraint
H-atom parameters constrained
Δρ_max_ = 0.21 e Å^−3^
Δρ_min_ = −0.19 e Å^−3^

### 

Data collection: *SMART* (Bruker, 2001 ▶); cell refinement: *SAINT* (Bruker, 2001 ▶); data reduction: *SAINT*; program(s) used to solve structure: *SHELXS97* (Sheldrick, 2008 ▶); program(s) used to refine structure: *SHELXL97* (Sheldrick, 2008 ▶); molecular graphics: *SHELXTL* (Sheldrick, 2008 ▶); software used to prepare material for publication: *SHELXTL*.

## Supplementary Material

Crystal structure: contains datablocks I, global. DOI: 10.1107/S1600536809043888/bt5079sup1.cif

Structure factors: contains datablocks I. DOI: 10.1107/S1600536809043888/bt5079Isup2.hkl

Additional supplementary materials:  crystallographic information; 3D view; checkCIF report

Enhanced figure: interactive version of Fig. 2

## Figures and Tables

**Fig. 1.**
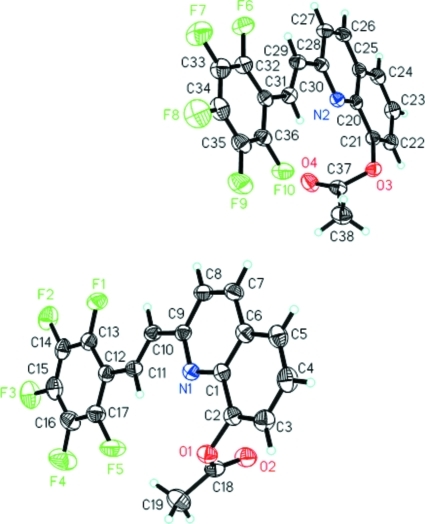
View of the two molecules in the asymmetric unit of the title compound.

## supplementary crystallographic information

### Comment

Herein, we report the crystal structure of (E)-2-[2-(pentafluorophenyl)ethenyl]-8-acetoxyquinoline, which was prepared via a reaction of 2-methyl-8-hydroxyquinaldine with pentafluorobenzaldehyde according to the procedure reported by Zeng *et al.* (2006). The title compound crystallizes with two almost identical molecules in asymmetric unit (Fig. 1.). The pentafluorobenzene ring is essentially coplanar with quinoline ring.

### Experimental

To a solution of 8-hydroxyquinaldine(1.19 g, 7.5 mmol) in acetic anhydride (5 mL) was added pentafluorobenzaldehyde (1.47 g, 7.5 mmol). The mixture was heated under reflux for 14 h . After cooling down to room temperature, it was subsequently poured into ice water (50 mL) and stirred overnight. The yellow solid obtained was filtered and washed with water. The solid residue was recrystallized from CH_2_Cl_2_ to afford the title compound   (2.13 g, 75%) mp 129-131 °C, ^1^H NMR (CDCl_3_, 300 MHz): 8.18 (d, J=8.7 Hz, 1H), 7.85 (d, J=16.5 Hz, 1H), 7.70 (dd, J=1.6 Hz, J=7.8 Hz 1H), 7.67 (d, J=16.5 Hz, 1H), 7.55 (t, J=8.4 Hz, 1H), 7.53 (d, J=8.1 Hz, 1H), 7.48 (dd, J=1.6 Hz, J=7.6 Hz, 1H), 2.56(s, 3H); ^19^F NMR (CDCl_3_, 282 MHz): -141.35 to 141.41(2F, m), -154.35 to 154.50(1F,m), -162.32 to 162.50 (2F, m); IR (KBr, cm^-1^): 3056, 1717, 1584, 1512, 1423, 1275, 1128, 987, 878, 765, 710; EI-MS m/z:(%) 379.0 [M^+^,0.86], 338.0 [(M-61)^+^, 20], 337.0 [(M-62)^+^, 100]; Elemental analysis: found C: 59.97, H: 2.30, N: 3.50 calculated for C_19_H_10_F_5_NO_2_: C, 60.17; H, 2.66; N, 3.69 (%)

### Refinement

All H atoms were positioned geometrically and refined using a riding model (including free rotation about the ethanol C-C bond), with C-H = 0.93-0.96 Å and with *U*_iso_(H) = 1.2*U*_eq_(C) or with *U*_iso_(H) = 1.5*U*_eq_(C_methyl_). Due to the absence of anomalous scatterers, the absolute structure could not be determined and was arbitrarily set. Friedel pairs were merged.

### Crystal data

**Table:**

C_19_H_10_F_5_NO_2_	*F*(000) = 768
*M**_r_* = 379.28	*D*_x_ = 1.571 Mg m^−^^3^
Monoclinic, *P*2_1_	Mo *K*α radiation, λ = 0.71073 Å
*a* = 12.3149 (13) Å	Cell parameters from 3790 reflections
*b* = 8.6730 (9) Å	θ = 2.1–27.0°
*c* = 15.0491 (16) Å	µ = 0.14 mm^−^^1^
β = 93.786 (2)°	*T* = 293 K
*V* = 1603.8 (3) Å^3^	Prismatic, colorless
*Z* = 4	0.40 × 0.37 × 0.23 mm

### Data collection

**Table:**

Bruker SMART CCD area-detector diffractometer	3695 independent reflections
Radiation source: fine-focus sealed tube	2952 reflections with *I* > 2σ(*I*)
graphite	*R*_int_ = 0.041
φ and ω scans	θ_max_ = 27.0°, θ_min_ = 2.1°
Absorption correction: multi-scan (*SADABS*; Bruker, 2001)	*h* = −12→15
*T*_min_ = 0.946, *T*_max_ = 0.968	*k* = −10→11
9498 measured reflections	*l* = −18→19

### Refinement

**Table:**

Refinement on *F*^2^	Secondary atom site location: difference Fourier map
Least-squares matrix: full	Hydrogen site location: inferred from neighbouring sites
*R*[*F*^2^ > 2σ(*F*^2^)] = 0.043	H-atom parameters constrained
*wR*(*F*^2^) = 0.108	*w* = 1/[σ^2^(*F*_o_^2^) + (0.0647*P*)^2^] where *P* = (*F*_o_^2^ + 2*F*_c_^2^)/3
*S* = 0.98	(Δ/σ)_max_ = 0.001
3695 reflections	Δρ_max_ = 0.21 e Å^−^^3^
490 parameters	Δρ_min_ = −0.19 e Å^−^^3^
1 restraint	Extinction correction: *SHELXL97* (Sheldrick, 2008), Fc^*^=kFc[1+0.001xFc^2^λ^3^/sin(2θ)]^-1/4^
Primary atom site location: structure-invariant direct methods	Extinction coefficient: 0.0061 (13)

### Special details

**Table:**

Geometry. All esds (except the esd in the dihedral angle between two l.s. planes) are estimated using the full covariance matrix. The cell esds are taken into account individually in the estimation of esds in distances, angles and torsion angles; correlations between esds in cell parameters are only used when they are defined by crystal symmetry. An approximate (isotropic) treatment of cell esds is used for estimating esds involving l.s. planes.
Refinement. Refinement of F^2^ against ALL reflections. The weighted R-factor wR and goodness of fit S are based on F^2^, conventional R-factors R are based on F, with F set to zero for negative F^2^. The threshold expression of F^2^ > 2sigma(F^2^) is used only for calculating R-factors(gt) etc. and is not relevant to the choice of reflections for refinement. R-factors based on F^2^ are statistically about twice as large as those based on F, and R- factors based on ALL data will be even larger.

### Fractional atomic coordinates and isotropic or equivalent isotropic displacement parameters (Å^2^)

**Table:**

	*x*	*y*	*z*	*U*_iso_*/*U*_eq_	
F1	0.33611 (15)	−0.0821 (3)	0.65824 (12)	0.0664 (6)	
F2	0.49061 (15)	−0.2657 (3)	0.73041 (16)	0.0803 (7)	
F3	0.49694 (18)	−0.3382 (3)	0.90485 (18)	0.0946 (8)	
F4	0.3463 (2)	−0.2163 (4)	1.00830 (16)	0.1119 (10)	
F5	0.19062 (18)	−0.0336 (3)	0.93763 (12)	0.0867 (8)	
F6	0.36601 (15)	0.4554 (3)	0.08205 (11)	0.0660 (6)	
F7	0.19161 (18)	0.2788 (4)	0.07778 (15)	0.0931 (8)	
F8	0.06916 (18)	0.2615 (4)	0.22027 (18)	0.1004 (9)	
F9	0.12930 (17)	0.4170 (3)	0.37066 (14)	0.0825 (7)	
F10	0.30608 (16)	0.5948 (3)	0.37770 (11)	0.0660 (6)	
O1	−0.16350 (15)	0.2719 (3)	0.84497 (13)	0.0506 (5)	
O2	−0.02488 (19)	0.4204 (3)	0.89609 (14)	0.0641 (6)	
O3	0.61824 (15)	1.0012 (3)	0.43067 (12)	0.0470 (5)	
O4	0.67283 (19)	0.7628 (3)	0.46897 (16)	0.0686 (7)	
N1	−0.01615 (17)	0.2314 (3)	0.71999 (14)	0.0423 (5)	
N2	0.60340 (17)	0.8523 (3)	0.27113 (14)	0.0394 (5)	
C1	−0.1009 (2)	0.3273 (4)	0.70004 (18)	0.0412 (6)	
C2	−0.1761 (2)	0.3553 (4)	0.76599 (19)	0.0458 (7)	
C3	−0.2632 (2)	0.4477 (5)	0.7503 (2)	0.0578 (8)	
H3	−0.3116	0.4635	0.7944	0.069*	
C4	−0.2803 (3)	0.5196 (5)	0.6673 (3)	0.0663 (10)	
H4	−0.3406	0.5831	0.6567	0.080*	
C5	−0.2108 (3)	0.4990 (4)	0.6018 (2)	0.0600 (9)	
H5	−0.2234	0.5483	0.5472	0.072*	
C6	−0.1195 (2)	0.4022 (4)	0.61722 (19)	0.0487 (7)	
C7	−0.0439 (3)	0.3736 (4)	0.55332 (19)	0.0532 (8)	
H7	−0.0529	0.4185	0.4972	0.064*	
C8	0.0428 (3)	0.2798 (4)	0.57407 (18)	0.0514 (7)	
H8	0.0943	0.2621	0.5327	0.062*	
C9	0.0542 (2)	0.2100 (4)	0.65838 (17)	0.0418 (6)	
C10	0.1487 (2)	0.1104 (4)	0.68194 (19)	0.0460 (7)	
H10	0.1991	0.0941	0.6396	0.055*	
C11	0.1649 (2)	0.0435 (4)	0.76047 (19)	0.0460 (7)	
H11	0.1112	0.0611	0.7999	0.055*	
C12	0.2539 (2)	−0.0531 (4)	0.79412 (19)	0.0454 (7)	
C13	0.3347 (2)	−0.1141 (4)	0.7447 (2)	0.0493 (7)	
C14	0.4153 (2)	−0.2109 (4)	0.7816 (2)	0.0560 (8)	
C15	0.4186 (3)	−0.2449 (5)	0.8695 (3)	0.0647 (9)	
C16	0.3431 (3)	−0.1861 (6)	0.9211 (2)	0.0691 (10)	
C17	0.2627 (3)	−0.0916 (5)	0.8837 (2)	0.0592 (9)	
C18	−0.0773 (2)	0.3068 (4)	0.90196 (19)	0.0505 (7)	
C19	−0.0608 (3)	0.1843 (6)	0.9706 (3)	0.0765 (11)	
H19A	−0.0231	0.2267	1.0229	0.115*	
H19B	−0.0185	0.1022	0.9477	0.115*	
H19C	−0.1302	0.1451	0.9856	0.115*	
C20	0.6904 (2)	0.9448 (3)	0.28986 (17)	0.0372 (6)	
C21	0.7023 (2)	1.0169 (4)	0.37360 (17)	0.0419 (6)	
C22	0.7883 (2)	1.1090 (4)	0.3970 (2)	0.0492 (7)	
H22	0.7945	1.1549	0.4529	0.059*	
C23	0.8684 (2)	1.1348 (4)	0.3357 (2)	0.0542 (8)	
H23	0.9275	1.1979	0.3517	0.065*	
C24	0.8601 (2)	1.0690 (4)	0.2543 (2)	0.0486 (7)	
H24	0.9138	1.0868	0.2149	0.058*	
C25	0.7707 (2)	0.9734 (4)	0.22833 (18)	0.0415 (7)	
C26	0.7552 (2)	0.9032 (4)	0.14474 (18)	0.0468 (7)	
H26	0.8052	0.9189	0.1020	0.056*	
C27	0.6678 (2)	0.8130 (4)	0.12635 (18)	0.0477 (7)	
H27	0.6563	0.7683	0.0704	0.057*	
C28	0.5935 (2)	0.7863 (4)	0.19247 (17)	0.0398 (6)	
C29	0.5021 (2)	0.6793 (4)	0.17519 (18)	0.0446 (7)	
H29	0.4894	0.6391	0.1182	0.053*	
C30	0.4372 (2)	0.6379 (4)	0.23760 (18)	0.0433 (6)	
H30	0.4531	0.6808	0.2936	0.052*	
C31	0.3443 (2)	0.5342 (4)	0.23035 (18)	0.0414 (6)	
C32	0.3102 (2)	0.4496 (4)	0.15604 (19)	0.0483 (7)	
C33	0.2199 (3)	0.3571 (5)	0.1518 (2)	0.0618 (9)	
C34	0.1585 (3)	0.3476 (5)	0.2244 (3)	0.0637 (9)	
C35	0.1887 (2)	0.4275 (5)	0.2996 (2)	0.0576 (8)	
C36	0.2797 (2)	0.5173 (4)	0.30251 (19)	0.0479 (7)	
C37	0.6063 (2)	0.8611 (4)	0.46929 (18)	0.0503 (7)	
C38	0.5006 (3)	0.8545 (5)	0.5107 (3)	0.0732 (11)	
H38A	0.4426	0.8487	0.4650	0.110*	
H38B	0.4919	0.9455	0.5459	0.110*	
H38C	0.4988	0.7650	0.5482	0.110*	

### Atomic displacement parameters (Å^2^)

**Table:**

	*U*^11^	*U*^22^	*U*^33^	*U*^12^	*U*^13^	*U*^23^
F1	0.0666 (11)	0.0732 (15)	0.0619 (11)	0.0130 (11)	0.0232 (9)	−0.0028 (11)
F2	0.0549 (11)	0.0711 (15)	0.1170 (17)	0.0146 (11)	0.0214 (11)	−0.0119 (13)
F3	0.0722 (13)	0.0779 (17)	0.130 (2)	0.0198 (13)	−0.0197 (13)	0.0159 (16)
F4	0.1202 (19)	0.142 (3)	0.0729 (14)	0.035 (2)	−0.0018 (13)	0.0403 (17)
F5	0.0912 (14)	0.114 (2)	0.0575 (11)	0.0362 (15)	0.0268 (10)	0.0183 (13)
F6	0.0671 (11)	0.0779 (16)	0.0541 (10)	−0.0180 (10)	0.0122 (8)	−0.0129 (10)
F7	0.0866 (15)	0.103 (2)	0.0887 (15)	−0.0428 (15)	−0.0011 (12)	−0.0268 (16)
F8	0.0693 (13)	0.107 (2)	0.1263 (19)	−0.0491 (15)	0.0166 (13)	−0.0048 (18)
F9	0.0727 (13)	0.0947 (19)	0.0836 (14)	−0.0132 (13)	0.0324 (11)	0.0185 (14)
F10	0.0714 (12)	0.0817 (16)	0.0459 (9)	−0.0095 (11)	0.0111 (8)	−0.0016 (10)
O1	0.0426 (10)	0.0584 (14)	0.0512 (11)	−0.0076 (10)	0.0065 (9)	0.0020 (11)
O2	0.0658 (13)	0.0701 (17)	0.0568 (13)	−0.0208 (13)	0.0067 (10)	−0.0049 (13)
O3	0.0470 (10)	0.0479 (13)	0.0477 (11)	0.0005 (10)	0.0149 (8)	−0.0014 (10)
O4	0.0649 (13)	0.0676 (17)	0.0754 (15)	0.0162 (14)	0.0199 (11)	0.0228 (14)
N1	0.0400 (11)	0.0435 (14)	0.0433 (12)	−0.0035 (11)	0.0014 (9)	0.0011 (11)
N2	0.0336 (11)	0.0426 (14)	0.0427 (12)	−0.0016 (10)	0.0073 (9)	0.0035 (10)
C1	0.0348 (13)	0.0412 (16)	0.0473 (14)	−0.0060 (12)	0.0003 (11)	0.0007 (13)
C2	0.0399 (14)	0.0467 (18)	0.0509 (16)	−0.0044 (13)	0.0025 (12)	0.0012 (14)
C3	0.0445 (16)	0.062 (2)	0.0677 (19)	0.0028 (16)	0.0081 (14)	−0.0006 (19)
C4	0.0514 (18)	0.062 (2)	0.084 (2)	0.0147 (18)	−0.0045 (16)	0.006 (2)
C5	0.0581 (18)	0.054 (2)	0.0669 (19)	0.0023 (17)	−0.0064 (16)	0.0072 (18)
C6	0.0465 (16)	0.0482 (19)	0.0506 (15)	−0.0069 (14)	−0.0030 (12)	0.0034 (14)
C7	0.0615 (18)	0.056 (2)	0.0423 (14)	−0.0057 (16)	0.0010 (13)	0.0055 (14)
C8	0.0571 (16)	0.057 (2)	0.0411 (14)	−0.0033 (16)	0.0089 (12)	−0.0011 (15)
C9	0.0426 (14)	0.0424 (17)	0.0407 (13)	−0.0046 (12)	0.0043 (11)	−0.0037 (13)
C10	0.0433 (15)	0.0485 (19)	0.0472 (15)	0.0000 (13)	0.0103 (12)	−0.0032 (13)
C11	0.0420 (14)	0.0492 (18)	0.0480 (15)	0.0015 (13)	0.0112 (11)	−0.0010 (14)
C12	0.0407 (14)	0.0419 (17)	0.0542 (16)	−0.0041 (13)	0.0080 (12)	−0.0003 (14)
C13	0.0440 (15)	0.0469 (19)	0.0577 (17)	−0.0060 (14)	0.0080 (13)	−0.0028 (15)
C14	0.0415 (16)	0.0430 (19)	0.084 (2)	0.0001 (14)	0.0062 (15)	−0.0094 (17)
C15	0.0505 (17)	0.050 (2)	0.092 (3)	0.0023 (16)	−0.0093 (17)	0.0047 (19)
C16	0.070 (2)	0.073 (3)	0.063 (2)	0.006 (2)	−0.0021 (17)	0.015 (2)
C17	0.0595 (18)	0.063 (2)	0.0555 (17)	0.0064 (17)	0.0108 (14)	0.0055 (17)
C18	0.0454 (15)	0.060 (2)	0.0470 (15)	0.0009 (16)	0.0123 (12)	−0.0009 (15)
C19	0.074 (2)	0.085 (3)	0.069 (2)	−0.002 (2)	−0.0049 (18)	0.018 (2)
C20	0.0337 (12)	0.0340 (15)	0.0442 (13)	0.0044 (11)	0.0042 (10)	0.0066 (12)
C21	0.0396 (13)	0.0425 (17)	0.0446 (14)	0.0012 (13)	0.0100 (11)	0.0041 (13)
C22	0.0486 (16)	0.0466 (18)	0.0525 (16)	−0.0019 (14)	0.0035 (13)	−0.0012 (14)
C23	0.0429 (15)	0.051 (2)	0.069 (2)	−0.0127 (14)	0.0024 (14)	0.0026 (17)
C24	0.0390 (15)	0.0490 (19)	0.0588 (17)	−0.0066 (14)	0.0099 (12)	0.0107 (15)
C25	0.0372 (13)	0.0403 (17)	0.0479 (15)	0.0009 (12)	0.0098 (11)	0.0090 (13)
C26	0.0461 (15)	0.0518 (19)	0.0442 (14)	−0.0007 (14)	0.0164 (12)	0.0086 (14)
C27	0.0511 (16)	0.054 (2)	0.0384 (14)	−0.0018 (15)	0.0076 (12)	0.0011 (14)
C28	0.0358 (13)	0.0426 (16)	0.0408 (13)	0.0018 (12)	0.0025 (10)	0.0051 (13)
C29	0.0404 (14)	0.0520 (19)	0.0409 (14)	0.0007 (13)	−0.0005 (11)	0.0027 (13)
C30	0.0374 (13)	0.0462 (17)	0.0458 (14)	0.0020 (12)	−0.0013 (11)	−0.0013 (13)
C31	0.0363 (12)	0.0411 (17)	0.0466 (14)	0.0019 (12)	0.0015 (11)	0.0088 (13)
C32	0.0415 (14)	0.053 (2)	0.0508 (15)	−0.0039 (14)	0.0026 (12)	−0.0020 (15)
C33	0.0545 (18)	0.065 (2)	0.0650 (19)	−0.0119 (17)	−0.0017 (15)	−0.0051 (18)
C34	0.0471 (17)	0.060 (2)	0.084 (2)	−0.0156 (17)	0.0017 (16)	0.0071 (19)
C35	0.0471 (16)	0.062 (2)	0.0647 (19)	−0.0013 (17)	0.0139 (14)	0.0155 (18)
C36	0.0452 (15)	0.053 (2)	0.0455 (15)	0.0037 (14)	0.0013 (12)	0.0069 (14)
C37	0.0486 (16)	0.060 (2)	0.0432 (15)	−0.0032 (16)	0.0080 (12)	0.0048 (14)
C38	0.066 (2)	0.079 (3)	0.079 (2)	−0.008 (2)	0.0320 (17)	0.010 (2)

### Geometric parameters (Å, °)

**Table:**

F1—C13	1.332 (4)	C12—C17	1.386 (4)
F2—C14	1.332 (4)	C12—C13	1.387 (4)
F3—C15	1.341 (4)	C13—C14	1.387 (5)
F4—C16	1.336 (4)	C14—C15	1.354 (5)
F5—C17	1.339 (4)	C15—C16	1.350 (5)
F6—C32	1.348 (3)	C16—C17	1.378 (5)
F7—C33	1.331 (4)	C18—C19	1.486 (5)
F8—C34	1.328 (4)	C19—H19A	0.9600
F9—C35	1.338 (3)	C19—H19B	0.9600
F10—C36	1.338 (4)	C19—H19C	0.9600
O1—C18	1.354 (3)	C20—C21	1.406 (4)
O1—C2	1.391 (4)	C20—C25	1.421 (3)
O2—C18	1.184 (4)	C21—C22	1.354 (4)
O3—C37	1.359 (4)	C22—C23	1.411 (4)
O3—C21	1.395 (3)	C22—H22	0.9300
O4—C37	1.182 (4)	C23—C24	1.349 (5)
N1—C9	1.323 (3)	C23—H23	0.9300
N1—C1	1.352 (4)	C24—C25	1.412 (4)
N2—C28	1.313 (4)	C24—H24	0.9300
N2—C20	1.354 (3)	C25—C26	1.399 (4)
C1—C6	1.411 (4)	C26—C27	1.344 (4)
C1—C2	1.423 (4)	C26—H26	0.9300
C2—C3	1.347 (4)	C27—C28	1.415 (4)
C3—C4	1.400 (5)	C27—H27	0.9300
C3—H3	0.9300	C28—C29	1.469 (4)
C4—C5	1.359 (5)	C29—C30	1.323 (4)
C4—H4	0.9300	C29—H29	0.9300
C5—C6	1.411 (5)	C30—C31	1.454 (4)
C5—H5	0.9300	C30—H30	0.9300
C6—C7	1.404 (4)	C31—C32	1.380 (4)
C7—C8	1.362 (5)	C31—C36	1.395 (4)
C7—H7	0.9300	C32—C33	1.370 (4)
C8—C9	1.405 (4)	C33—C34	1.371 (5)
C8—H8	0.9300	C34—C35	1.357 (5)
C9—C10	1.473 (4)	C35—C36	1.363 (5)
C10—C11	1.320 (4)	C37—C38	1.482 (4)
C10—H10	0.9300	C38—H38A	0.9600
C11—C12	1.443 (4)	C38—H38B	0.9600
C11—H11	0.9300	C38—H38C	0.9600
			
C18—O1—C2	117.6 (2)	H19A—C19—H19C	109.5
C37—O3—C21	117.1 (2)	H19B—C19—H19C	109.5
C9—N1—C1	117.5 (2)	N2—C20—C21	119.0 (2)
C28—N2—C20	118.1 (2)	N2—C20—C25	122.9 (2)
N1—C1—C6	123.8 (3)	C21—C20—C25	118.1 (2)
N1—C1—C2	118.6 (2)	C22—C21—O3	119.8 (2)
C6—C1—C2	117.6 (3)	C22—C21—C20	121.9 (2)
C3—C2—O1	120.1 (3)	O3—C21—C20	118.1 (2)
C3—C2—C1	121.8 (3)	C21—C22—C23	119.5 (3)
O1—C2—C1	117.8 (3)	C21—C22—H22	120.3
C2—C3—C4	119.5 (3)	C23—C22—H22	120.3
C2—C3—H3	120.3	C24—C23—C22	120.8 (3)
C4—C3—H3	120.3	C24—C23—H23	119.6
C5—C4—C3	121.6 (3)	C22—C23—H23	119.6
C5—C4—H4	119.2	C23—C24—C25	120.7 (3)
C3—C4—H4	119.2	C23—C24—H24	119.6
C4—C5—C6	119.5 (3)	C25—C24—H24	119.6
C4—C5—H5	120.3	C26—C25—C24	124.2 (2)
C6—C5—H5	120.3	C26—C25—C20	116.7 (3)
C7—C6—C5	123.4 (3)	C24—C25—C20	119.0 (3)
C7—C6—C1	116.6 (3)	C27—C26—C25	119.9 (2)
C5—C6—C1	120.0 (3)	C27—C26—H26	120.0
C8—C7—C6	119.6 (3)	C25—C26—H26	120.0
C8—C7—H7	120.2	C26—C27—C28	119.8 (3)
C6—C7—H7	120.2	C26—C27—H27	120.1
C7—C8—C9	119.6 (3)	C28—C27—H27	120.1
C7—C8—H8	120.2	N2—C28—C27	122.5 (3)
C9—C8—H8	120.2	N2—C28—C29	117.3 (2)
N1—C9—C8	122.8 (3)	C27—C28—C29	120.2 (3)
N1—C9—C10	117.2 (2)	C30—C29—C28	122.5 (3)
C8—C9—C10	120.0 (3)	C30—C29—H29	118.7
C11—C10—C9	122.9 (3)	C28—C29—H29	118.7
C11—C10—H10	118.5	C29—C30—C31	128.6 (3)
C9—C10—H10	118.5	C29—C30—H30	115.7
C10—C11—C12	129.5 (3)	C31—C30—H30	115.7
C10—C11—H11	115.2	C32—C31—C36	114.6 (3)
C12—C11—H11	115.2	C32—C31—C30	125.8 (2)
C17—C12—C13	114.6 (3)	C36—C31—C30	119.5 (3)
C17—C12—C11	119.3 (3)	F6—C32—C33	116.1 (3)
C13—C12—C11	126.1 (3)	F6—C32—C31	120.4 (3)
F1—C13—C14	117.5 (3)	C33—C32—C31	123.5 (3)
F1—C13—C12	120.0 (3)	F7—C33—C32	120.3 (3)
C14—C13—C12	122.5 (3)	F7—C33—C34	120.6 (3)
F2—C14—C15	120.7 (3)	C32—C33—C34	119.1 (3)
F2—C14—C13	119.5 (3)	F8—C34—C35	120.4 (3)
C15—C14—C13	119.8 (3)	F8—C34—C33	119.6 (3)
F3—C15—C16	120.3 (4)	C35—C34—C33	119.9 (3)
F3—C15—C14	119.6 (3)	F9—C35—C34	119.8 (3)
C16—C15—C14	120.2 (3)	F9—C35—C36	120.3 (3)
F4—C16—C15	121.0 (3)	C34—C35—C36	119.8 (3)
F4—C16—C17	119.4 (3)	F10—C36—C35	117.9 (3)
C15—C16—C17	119.6 (3)	F10—C36—C31	119.1 (3)
F5—C17—C16	117.6 (3)	C35—C36—C31	123.0 (3)
F5—C17—C12	119.1 (3)	O4—C37—O3	123.2 (3)
C16—C17—C12	123.3 (3)	O4—C37—C38	127.0 (3)
O2—C18—O1	123.1 (3)	O3—C37—C38	109.7 (3)
O2—C18—C19	126.6 (3)	C37—C38—H38A	109.5
O1—C18—C19	110.3 (3)	C37—C38—H38B	109.5
C18—C19—H19A	109.5	H38A—C38—H38B	109.5
C18—C19—H19B	109.5	C37—C38—H38C	109.5
H19A—C19—H19B	109.5	H38A—C38—H38C	109.5
C18—C19—H19C	109.5	H38B—C38—H38C	109.5
			
C9—N1—C1—C6	1.4 (4)	C28—N2—C20—C21	179.8 (3)
C9—N1—C1—C2	−178.4 (3)	C28—N2—C20—C25	0.1 (4)
C18—O1—C2—C3	−115.3 (3)	C37—O3—C21—C22	−112.4 (3)
C18—O1—C2—C1	71.0 (4)	C37—O3—C21—C20	72.5 (3)
N1—C1—C2—C3	−179.1 (3)	N2—C20—C21—C22	179.0 (3)
C6—C1—C2—C3	1.1 (5)	C25—C20—C21—C22	−1.2 (4)
N1—C1—C2—O1	−5.4 (4)	N2—C20—C21—O3	−6.0 (4)
C6—C1—C2—O1	174.7 (3)	C25—C20—C21—O3	173.8 (2)
O1—C2—C3—C4	−174.1 (3)	O3—C21—C22—C23	−174.5 (3)
C1—C2—C3—C4	−0.6 (5)	C20—C21—C22—C23	0.4 (5)
C2—C3—C4—C5	−0.2 (6)	C21—C22—C23—C24	0.1 (5)
C3—C4—C5—C6	0.4 (6)	C22—C23—C24—C25	0.4 (5)
C4—C5—C6—C7	179.7 (3)	C23—C24—C25—C26	178.8 (3)
C4—C5—C6—C1	0.2 (5)	C23—C24—C25—C20	−1.2 (5)
N1—C1—C6—C7	−0.3 (5)	N2—C20—C25—C26	1.3 (4)
C2—C1—C6—C7	179.6 (3)	C21—C20—C25—C26	−178.4 (3)
N1—C1—C6—C5	179.3 (3)	N2—C20—C25—C24	−178.6 (3)
C2—C1—C6—C5	−0.9 (5)	C21—C20—C25—C24	1.6 (4)
C5—C6—C7—C8	179.3 (3)	C24—C25—C26—C27	179.5 (3)
C1—C6—C7—C8	−1.2 (5)	C20—C25—C26—C27	−0.5 (4)
C6—C7—C8—C9	1.5 (5)	C25—C26—C27—C28	−1.6 (5)
C1—N1—C9—C8	−1.2 (4)	C20—N2—C28—C27	−2.4 (4)
C1—N1—C9—C10	177.6 (3)	C20—N2—C28—C29	176.5 (2)
C7—C8—C9—N1	−0.3 (5)	C26—C27—C28—N2	3.2 (5)
C7—C8—C9—C10	−179.0 (3)	C26—C27—C28—C29	−175.7 (3)
N1—C9—C10—C11	0.1 (5)	N2—C28—C29—C30	−6.2 (5)
C8—C9—C10—C11	178.9 (3)	C27—C28—C29—C30	172.7 (3)
C9—C10—C11—C12	−178.3 (3)	C28—C29—C30—C31	179.9 (3)
C10—C11—C12—C17	170.0 (3)	C29—C30—C31—C32	4.7 (5)
C10—C11—C12—C13	−10.0 (6)	C29—C30—C31—C36	−173.8 (3)
C17—C12—C13—F1	−178.5 (3)	C36—C31—C32—F6	−179.7 (3)
C11—C12—C13—F1	1.5 (5)	C30—C31—C32—F6	1.8 (5)
C17—C12—C13—C14	2.4 (5)	C36—C31—C32—C33	0.7 (5)
C11—C12—C13—C14	−177.5 (3)	C30—C31—C32—C33	−177.9 (3)
F1—C13—C14—F2	0.9 (5)	F6—C32—C33—F7	0.2 (5)
C12—C13—C14—F2	180.0 (3)	C31—C32—C33—F7	179.9 (3)
F1—C13—C14—C15	178.9 (3)	F6—C32—C33—C34	−179.1 (3)
C12—C13—C14—C15	−2.0 (5)	C31—C32—C33—C34	0.5 (6)
F2—C14—C15—F3	−1.8 (6)	F7—C33—C34—F8	−1.1 (6)
C13—C14—C15—F3	−179.8 (3)	C32—C33—C34—F8	178.2 (3)
F2—C14—C15—C16	178.6 (4)	F7—C33—C34—C35	179.7 (4)
C13—C14—C15—C16	0.6 (6)	C32—C33—C34—C35	−1.0 (6)
F3—C15—C16—F4	1.6 (7)	F8—C34—C35—F9	1.5 (6)
C14—C15—C16—F4	−178.8 (4)	C33—C34—C35—F9	−179.4 (4)
F3—C15—C16—C17	−179.5 (4)	F8—C34—C35—C36	−179.0 (3)
C14—C15—C16—C17	0.1 (7)	C33—C34—C35—C36	0.1 (6)
F4—C16—C17—F5	0.1 (6)	F9—C35—C36—F10	−0.9 (5)
C15—C16—C17—F5	−178.9 (4)	C34—C35—C36—F10	179.6 (3)
F4—C16—C17—C12	179.4 (4)	F9—C35—C36—C31	−179.3 (3)
C15—C16—C17—C12	0.5 (7)	C34—C35—C36—C31	1.2 (5)
C13—C12—C17—F5	177.6 (3)	C32—C31—C36—F10	−180.0 (3)
C11—C12—C17—F5	−2.4 (5)	C30—C31—C36—F10	−1.3 (4)
C13—C12—C17—C16	−1.7 (6)	C32—C31—C36—C35	−1.6 (5)
C11—C12—C17—C16	178.3 (4)	C30—C31—C36—C35	177.1 (3)
C2—O1—C18—O2	14.2 (4)	C21—O3—C37—O4	13.1 (4)
C2—O1—C18—C19	−166.2 (3)	C21—O3—C37—C38	−167.5 (3)
